# Validation data of parallel 3D surface-borehole electromagnetic forward modeling

**DOI:** 10.1016/j.dib.2020.105209

**Published:** 2020-01-30

**Authors:** Chong Liu, LiZhen Cheng, Bahman Abbassi

**Affiliations:** Université du Québec en Abitibi-Témiscamingue, 445 boul. de l’Université, Rouyn-Noranda, QC, J9X 5E4, Canada

**Keywords:** Surface-borehole TEM forward modeling, Edge-based finite element, Parallelization, Multiple meshes, Computational cost

## Abstract

Forward modeling of geophysical electromagnetic fields over large three-dimensional volumes is a heavy computational task that demands effective accelerating strategies. As a solution to this computational challenge, a hybrid parallel computing algorithm with multiple meshes has been previously proposed for 3D forward modeling of time-domain electromagnetic (TEM) fields by Liu et al. (2019). MPI and OpenMP were used for parallel computing and multiple meshes for optimizing the design of the geological model and frequencies used in forward modeling. The data presented in this paper offers complementary information on the calculation of the different components, such as the model discretization with regular or multiple meshes, the parallel computing with even or uneven modes, and an example of 3D TEM forward modeling through the proposed algorithm.

**Table:** Specifications Table

Subject	Geophysics
Specific subject area	Parallel computing in TEM forward modeling
Type of data	Table, Figure, Text
How data were acquired	Numerical simulation
Data format	Raw, Analyzed
Parameters for data collection	The codes are compiled with the Intel Parallel Studio XE 2016 and Visual Studio 2015 software, with 8–16 processors, and 2 to 3 meshes. TEM signals are simulated for a 200 × 200 m (Tx) current loop (1A), and a square waveform (100 ms on-time, and 1000 ms off-time). The receivers (Rx) record the TEM signals in 5 boreholes.
Description of data collection	Data come from the TEM forward modeling, based on a geological model and designed measurement system's configuration.
Data source location	Université du Québec en Abitibi-Témiscamingue
Data accessibility	Repository name: Mendeley Data Data identification number: file-7a93f854-d562-4ecc-98d7-c3c937fb4a20 Direct URL to data: https://doi.org/10.17632/zjbbtcgbk7.3
Related research article	Liu, C., Cheng, L., and Abbassi, B., 2019. 3D parallel surface-borehole TEM forward modeling with multiple meshes. Journal of Applied Geophysics, p.103916. https://doi.org/10.1016/j.jappgeo.2019.103916

**Table:** 

**Value of the data** • Forward modeling helps to test different geological scenarios and to study the significance of geophysical exploration methods for specific geological settings. • Geophysicists who are looking for an example of TEM field survey design with high performance and quick TEM forward modeling, benefit from data presented here. • The present data can be used as a benchmark for future validations and comparisons. • The present data and methods can be used for further inverse modeling developments.

## 1 Data

This paper describes the application of a parallelized code for the surface-borehole TEM forward modeling. The Loki code [1,2] is parallelized with MPI and OpenMP with even and uneven modes and multiple meshes for 3D model discretization [3].

**Fig. 1:**
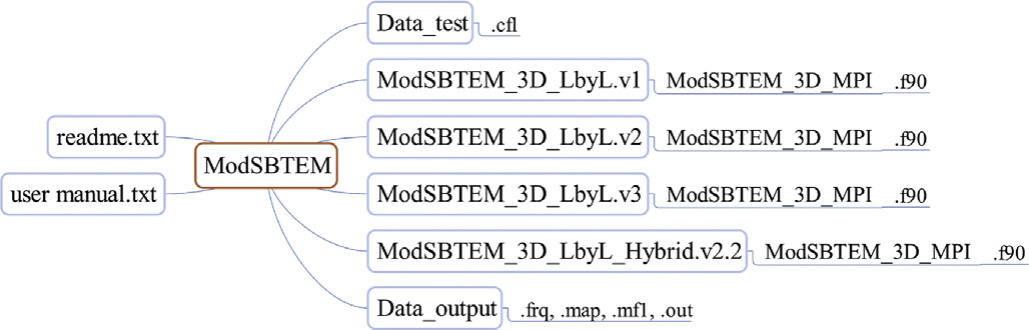
Organization of the datasets and the codes.

All data are organized in one folder, named “ModSBTEM”, with six subfolders (Fig. 1), including all the source codes and the input/output data used in the forward modeling. Detailed information can be found in the “readme” file. In the subfolder “ModSBTEM_3D_LbyL.v1”, regular meshes are used with even mode parallelization, i.e., the tasks are assigned to the processors evenly. In the subfolder “ModSBTEM_3D_LbyL.v2”, regular meshes are also used with uneven mode parallelization, i.e., the tasks are assigned to the processors randomly. In the subfolder “ModSBTEM_3D_LbyL.v3”, the forward modeling is parallelized with uneven mode, and the 3D volume is discretized by multiple meshes. In the subfolder “ModSBTEM_3D_LbyL_Hybrid.v2.2”, the forward modeling is parallelized with hybrid MPI and OpenMP.

All codes are compiled with the programming language ANSI Standard Fortran 95, running with the Visual Studio and Intel Parallel Studio XE. The file ‘user manual.txt’ describes the main information and data format used in the codes. Also, each subfolder contains a note file indicating the main structures of the codes.

**Fig. 2:**
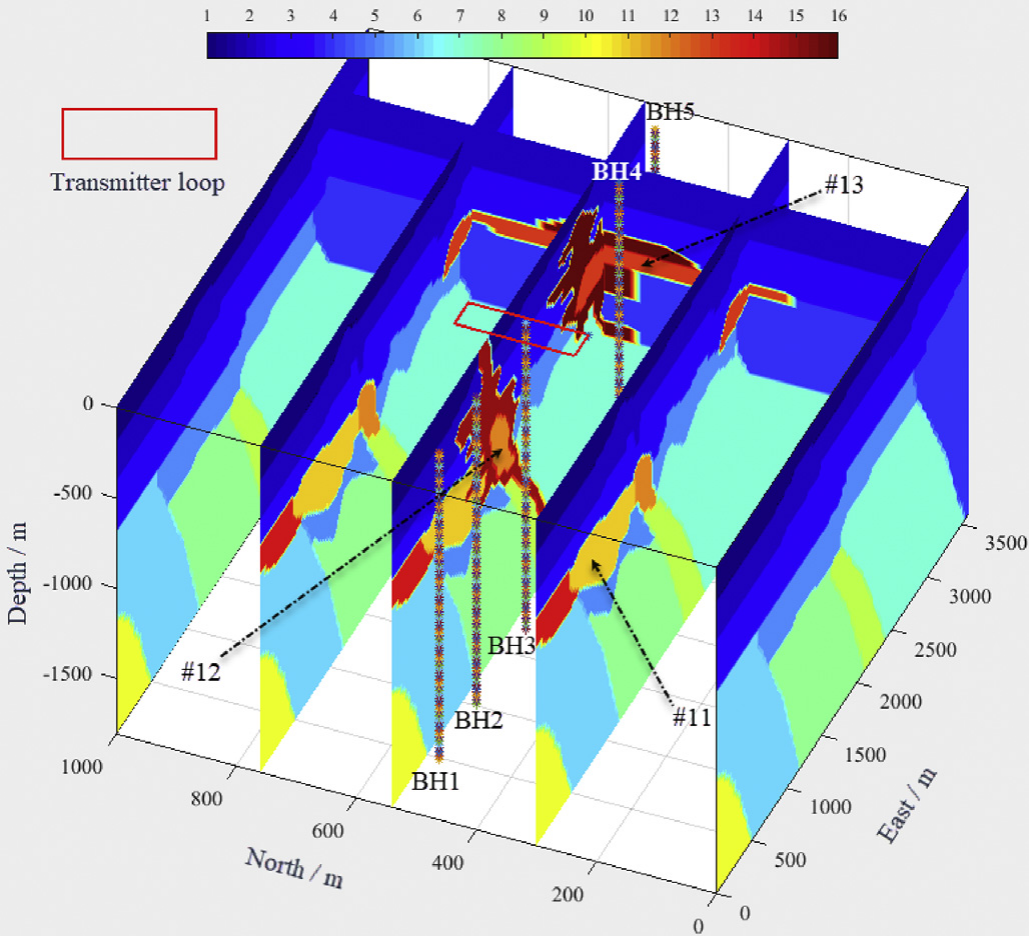
Complex synthetic model and the locations of the transmitter and boreholes, the numbers on the color bar denote the rock types.

**Fig. 3:**
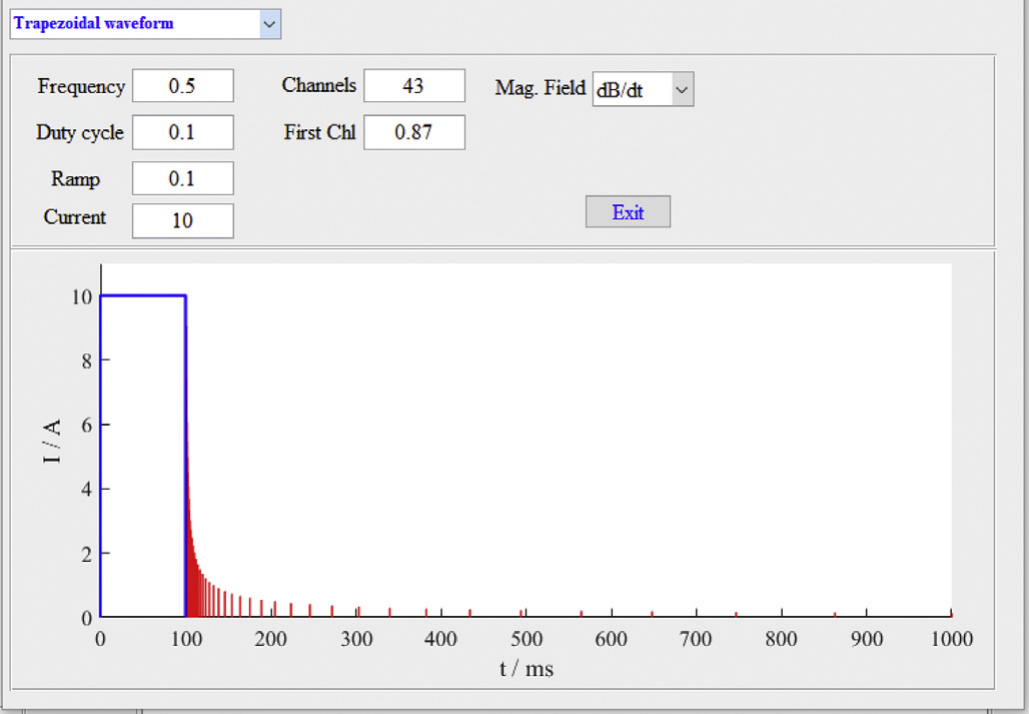
Transmitter waveform.

**Fig. 4:**
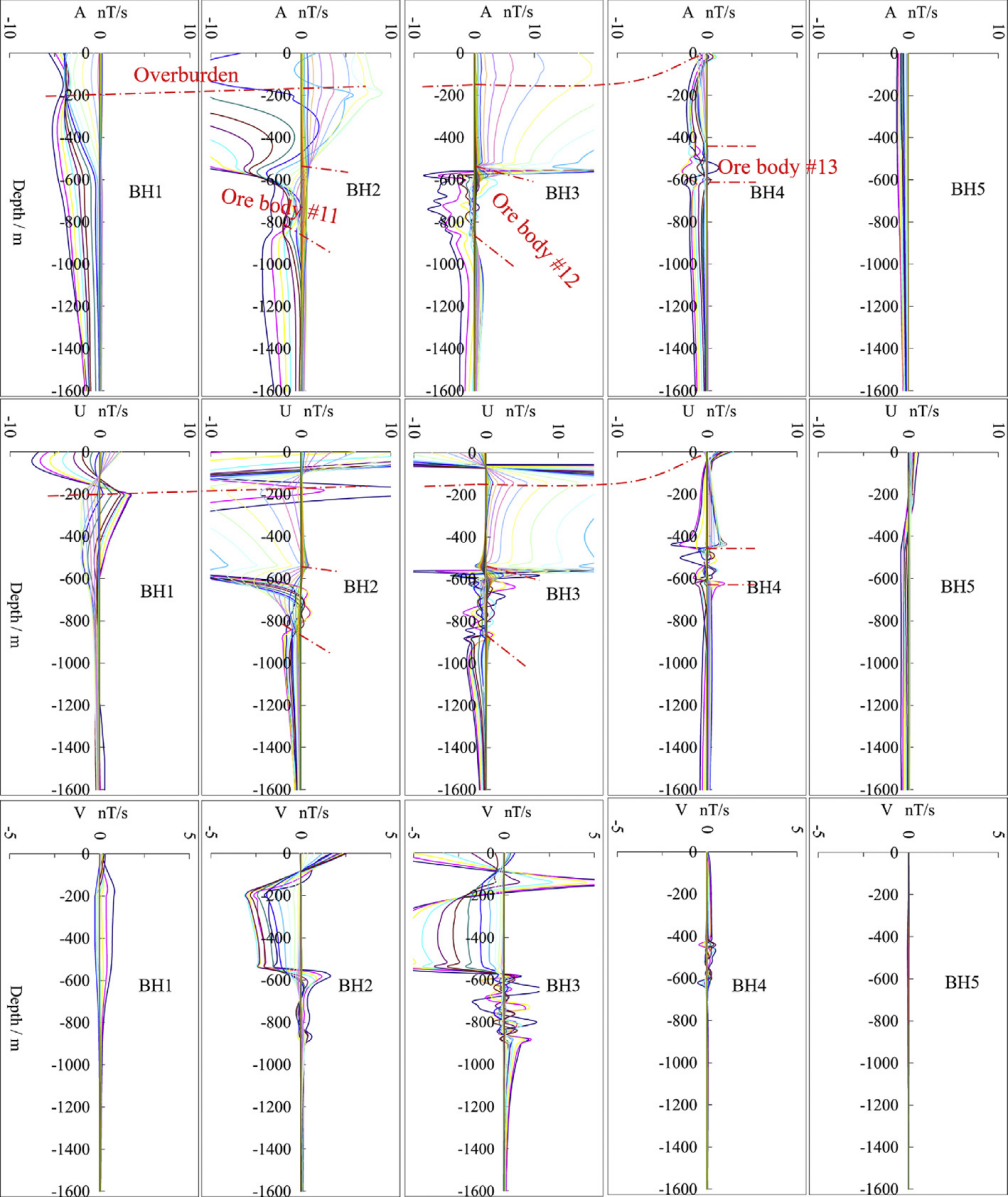
The responses (A, U, V components) of the model in five boreholes (BH1, BH2, BH3, BH4, and BH5).

The forward modeling process is shown through an example. Fig. 2 describes the survey configuration, including a transmitter loop with an array of TEM receivers in five boreholes within a 3D earth model. The main transmitter waveform is trapezoidal with a ramp (Fig. 3). The geology and physical properties of lithologies are based on a typical geological environment in the Abitibi greenstone belt of Canada (Table 1). The surface-borehole TEM responses generated by the parallelized code in five boreholes are illustrated in Fig. 4.

**Table 1:** Electric resistivity of rocks.

Rock symbols	Resistivity/Ω.m
#1	50 (overburden)
#2, #6, #14	2000 (sandstone/quartzite)
#3	1000 (mudstone)
#4, #10	90 000 (granite)
#5	3000 (paleo-valley)
#7, #8	60 000 (wet gneiss)
#9, #15, #16	2000 (silicified zone)
#11	5 (semi-metallic)
#12, #13	0.1 (Mono/Poly-metallic)

## 2 Experimental design, materials, and methods

### 2.1 Design

It is well known that the number of physical processors limits the speedup of parallel computing [3]. A fine mesh in the early time and a coarser mesh in the late time is proved as an efficient strategy to enhance the performance of parallel computing. We assign each frequency its effective region and mesh strategy in the frequency-domain simulation, and then the frequency-domain response is transformed into the time domain. In order to illustrate how to integrate multiple meshes into 3D modeling, we deployed this strategy in an example in the next section.

### 2.2 Illustration of the method

This example aims to simulate the surface-borehole TEM response of the ore bodies (lithologies #11, #12, and #13) hidden in a complex geological environment (Fig. 2). The template geological model was constructed from 45 cross-sections inside a 3D volume of 3.6 km (N–S) by 1 km (E-W) by 1.8 km (depth). Since the TEM response results from the coupling of the emitted electromagnetic field and the geological model, Fig. 3 illustrates the parameters of the primary electromagnetic field generated by a transmitter loop with a trapezoidal waveform. The center of the transmitter loop (200 m × 200 m) is placed on the surface at the point (1500 m, 500 m) in the model space. Five boreholes (Fig. 2) are along the N–S direction, which are BH1 (330 m, 500 m), BH2 (860 m, 500 m), BH3 (1560 m, 500 m), BH4 (2900 m, 500 m), and BH5 (3400 m, 500 m). TEM receivers in five boreholes measure the changes in the induced electromagnetic field due to the physical property variations (Table 1). Fig. 4 illustrates the TEM forward modeling responses from the 3D parallelized code.
